# Associations between multidimensional social support and satisfaction with community mental health services: evidence from China with heterogeneity analysis

**DOI:** 10.3389/fpubh.2026.1839534

**Published:** 2026-06-02

**Authors:** Haitao Li, Haibo Li, Feiyue Liu

**Affiliations:** 1 School of Public Administration and Law, Hunan Agricultural University, Changsha, China; 2 School of Economics and Management, Changsha University, Changsha, China

**Keywords:** China, heterogeneity analysis, patients with mental disorders, satisfaction with community mental health services, social support

## Abstract

**Background:**

Mental health presents a significant global public health challenge. China is transitioning toward a community-based mental health service model; however, service quality and satisfaction remain constrained by an overreliance on medical-technical solutions and insufficient attention to broader psychosocial factors. The mechanisms through which different dimensions of social support are associated with service satisfaction, and their variation across subgroups, are poorly understood. This study aims to investigate the impact of multidimensional social support on patients with mental disorders (PMD)’ satisfaction with community mental health services (SCMHS) and to examine the heterogeneity of these effects across gender and age groups.

**Methods:**

Drawing on social support theory, we constructed an analytical framework encompassing emotional, technical, and economic support dimensions. Field survey data were collected from three Chinese cities (Shanghai, Changsha, and Liuzhou) representing eastern, central, and western regions. A probit regression model was employed for empirical analysis, and robustness was tested using propensity score matching (PSM), alternative explanatory variables, and alternative regression models.

**Results:**

Social support is significantly and positively associated with SCMHS. All three dimensions—emotional (most prominent), technical, and economic support—exhibit significant positive associations. Heterogeneity analysis reveals that: (1) the positive association between social support and SCMHS is stronger for male patients than for females; (2) older adult patients are more strongly associated with economic support, whereas younger groups show stronger associations with emotional and technical support.

**Conclusion:**

The findings suggest that improving SCMHS may require an integrated support model that combines psychosocial care with medical interventions. Policy interventions should move beyond generalized supply toward precision matching, tailoring services to the specific needs of different demographic subgroups to enhance satisfaction and promote inclusive mental health governance.

## Introduction

Since the World Health Organization (WHO) Constitution in 1946 explicitly defined health as a state of complete physical, mental, and social well-being, mental health has become a core issue in global public health. According to the WHO’s *World Mental Health Report* published in 2022, approximately one billion people worldwide were living with mental disorders as of 2019, accounting for one-seventh of the global population. The substantial social burden and economic costs associated with mental illnesses make them a severe global challenge.

As a rapidly developing country undergoing significant social transition, China faces increasingly prominent mental health issues alongside its economic growth. The lifetime prevalence of mental disorders in China is 16.57%, and the number of affected individuals continues to rise annually (1). However, treatment-seeking behavior remains low—only 9.5% of people with depression have received treatment from healthcare facilities (2)—highlighting a substantial gap in service accessibility and quality. Mental illnesses are characterized by prolonged duration, high relapse rates, and significant risk of disability (3). Coupled with a large patient population, these challenges exacerbate the imbalance between supply and demand of medical resources and contribute to secondary harms such as institutionalization syndrome (4).

In response, China has been gradually transitioning its mental health service system toward a community-based model since the 1980s, undergoing a complex process of “Communitization- Institutionalization - Re-communitization” (5). In recent years, the government has introduced a series of policies to promote community-based rehabilitation for mental disorders. For instance, the “*Healthy China 2030*” *Initiative* in 2016 explicitly called for “comprehensive advancement of community-based rehabilitation services for mental disorders,” and the 2022 “Integration Action for Mental Health” further incorporated community mental health rehabilitation into the national mental health prevention and treatment system. International evidence suggests that community-based service models offer significant advantages in facilitating patient recovery, improving quality of life, and reducing social burden, and have become the mainstream approach in many countries (6, 7).

Nevertheless, the overall quality of community-based mental health services in China remains considerably constrained by multi-level systemic barriers. Existing research has predominantly focused on medical-technical dimensions, identifying structural bottlenecks such as unequal resource distribution (8, 9), shortage of specialized professionals (10, 11), and inadequate infrastructure and management systems (12–14). While these findings are valuable, it is essential to recognize that mental health is inherently a complex issue spanning biological, psychological, and social dimensions. Relying solely on medical-technical improvements is insufficient for achieving systemic solutions. Patient recovery and service satisfaction depend not only on professional interventions but are also profoundly shaped by emotional support, social integration, economic security, and institutional coordination (15, 16).

Furthermore, the existing literature exhibits two major limitations. First, few studies systematically compare the differential mechanisms through which various types of social support (e.g., emotional vs. instrumental support) are associated with satisfaction with mental health services. Second, there is a general neglect of heterogeneity across subgroups—such as variations in social support needs and service responsiveness among patients of different genders and age groups. Although prior research has indicated gender-based (17) and age-based (18, 19) disparities in mental health status and perceived social support, how these differences relate to satisfaction with community mental health services remains underexplored. These research gaps limit the precision and targeting of policy interventions, hindering the shift from “generalized supply” to “precision matching.”

To address these gaps, this study employs a social support theoretical framework focusing on three core dimensions—emotional support, technical support, and economic support—to systematically analyze how social support is associated with satisfaction with community mental health services (SCMHS). Using field survey data from three Chinese cities and applying a probit regression model, this research examines both the overall relationship between social support and SCMHS and the heterogeneity across demographic (e.g., gender, age) subgroups. The theoretical contribution of this study lies in proposing and validating a three-dimensional support–satisfaction analytical framework, thereby expanding the explanatory scope of social support theory in the context of community mental health services and enriching research perspectives and empirical evidence in the field. On a practical level, the empirical findings grounded in China’s context provide a scientific basis for transitioning community mental health services from “scale coverage” to “quality improvement,” and offer targeted policy insights for constructing a stratified, classified, and precisely-adapted mental health governance system.

### Theoretical framework and research hypotheses

#### Social support theory

Social Support Theory originated at the intersection of social psychology, sociology, and health sciences, aiming to elucidate the mechanisms through which social networks are associated with individual health and well-being. Cobb (20) first defined it as “an individual’s perceived care, respect, and assistance from others” and empirically demonstrated its close relationship with psychological health. Since then, the theory has been widely applied in mental health research, with numerous studies consistently highlighting the positive role of social support in psychological adaptation and disease rehabilitation (21).

The multidimensional nature of social support has been well recognized. In early research, Cobb (20) and Lin et al. (22) categorized it into instrumental support and emotional support, finding significantly higher risks of depression among populations lacking such support. House (23) further proposed a four-dimensional model encompassing emotional support, appraisal support, informational support, and instrumental support, emphasizing the central role of emotional support. Thoits (24) distinguished between formal support (from professional institutions) and informal support (from family and friend networks) based on the source of support. In the Chinese context, Xiao (25) developed the Social Support Rating Scale, which includes three dimensions: objective support, subjective support, and support utilization, forming an important foundation for domestic research. Subsequent scholars, such as Qiu et al. (26), differentiated between main effect and buffering effect mechanisms; Wei et al. (27) focused on instrumental and teacher support; while Shen et al. (28) examined the role of functional support, financial assistance, and other specific types of support among older adults.

Despite variations in dimensional classifications, there is a consensus in academia that social support promotes mental health through multiple pathways. Integrating existing theoretical achievements with the context of community mental health services in China, this study categorizes social support associated with SCMHS into three core dimensions:

- Emotional Support: Service providers (e.g., community psychiatric nurses, psychological counselors) enhance patients’ and their families’ psychological trust and security through empathy, respect, and care (29, 30).
- Technical Support: Refers to professional services such as diagnosis, medication, psychological intervention, and rehabilitation training provided by the government, community health service centers, or clinics (11, 13).
- Economic Support: Includes affordability measures such as medical expense reimbursement, reductions, and subsidies provided by government and community organizations (31).

These three dimensions are conceptually distinct: emotional support operates through psychological and affective pathways (e.g., trust-building, stigma reduction); technical support functions through clinical and rehabilitative pathways (e.g., symptom control, functional recovery); and economic support works through structural and resource-based pathways (e.g., financial barrier reduction, service accessibility). Understanding these distinctions is essential for examining how each dimension is uniquely associated with SCMHS.

#### Social support and SCMHS

**Figure 1:**
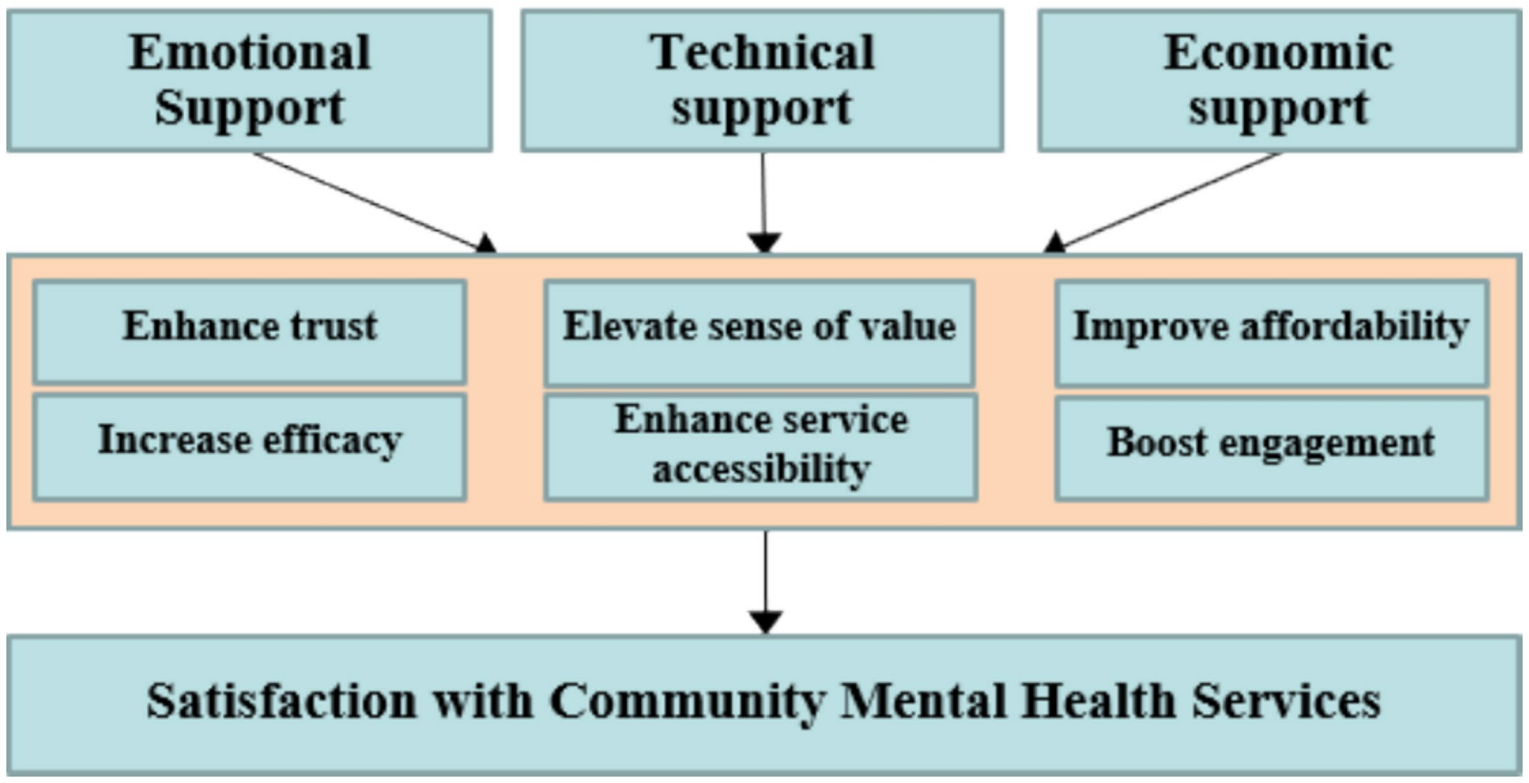
Theoretical analysis framework.

Driven by the global deinstitutionalization movement and the biopsychosocial medical model, community mental health services have become a core component of the mental disorder care system due to their advantages in promoting social integration and providing continuous support (32–35). Satisfaction with community mental health services (SCMHS) refers to the subjective evaluation by patients and their families regarding community-based prevention, treatment, and rehabilitation services. It not only directly influences treatment adherence and rehabilitation outcomes but also serves as a key indicator for measuring service quality and system effectiveness (36). As a complex construct, SCMHS is associated with a multidimensional support system. Based on social support theory and service quality models, this study constructs a “three-dimensional support–satisfaction” mechanism framework to examine how emotional, technical, and economic support are associated with SCMHS (Figure 1).

#### Emotional support and SCMHS

Emotional support is positively associated with SCMHS through pathways involving trust-building, stigma reduction, and enhanced self-efficacy. First, an empathetic and respectful service attitude helps establish a solid therapeutic alliance and increases patients’ trust. Such trust not only significantly improves overall satisfaction with services but also helps buffer the negative impact of technical deficiencies. Second, emotional support is associated with alleviated psychological stress and stigma among patients and their families. Stigma and discrimination have long been major concerns for individuals with mental disorders and their relatives (12, 37). Emotional support provides a safe channel for emotional expression and a psychological buffer, which may reduce mental pressure on both patients and families (38). Individuals who perceive higher levels of support tend to exhibit enhanced self-efficacy and are more likely to actively engage in their recovery process, which corresponds with higher satisfaction. Furthermore, supportive relationships may help restore hope and self-worth, thereby relating to improved treatment response and satisfaction (39). Based on this analysis, we propose:

*H1*: Emotional support has a positive association with PMD’ SCMHS.

#### Technical support and SCMHS

Technical support is positively associated with satisfaction with CMHS, potentially through enhanced symptom control and rehabilitation efficacy. Professional services such as standardized medication, psychological interventions, and rehabilitation training can directly alleviate symptoms and restore social functioning (11). Meanwhile, through health education and social skills training, patients’ self-management abilities may be improved (12, 40). This transfer of knowledge and skills may strengthen patients’ sense of control over their lives, thereby elevating their evaluation of the services. Optimized service processes and coordinated management further support intervention effectiveness and continuity (13, 41). Studies indicate that the scientific rigor and professionalism of rehabilitation plans significantly affect mental health outcomes (42, 43). When patients perceive tangible positive changes resulting from the services, their satisfaction tends to increase substantially. Based on this analysis, we propose:

*H2*: Technical support has a positive association with PMD’ SCMHS.

#### Economic support and SCMHS

Economic support is positively associated with SCMHS, potentially through reducing financial burden and improving service accessibility. Mental disorders are often associated with high treatment costs, which can easily lead to “catastrophic health expenditures” for families (44, 45). Financial aid, fee reductions, and health insurance policies may alleviate economic pressure and improve patients’ psychological well-being, thereby relating to higher service satisfaction. Moreover, economic support may lowers barriers to accessing services, ensuring that patients receive necessary interventions continuously and avoiding treatment discontinuation and relapse (46–48). This may enables emotional and technical support to yield long-term effects, thereby sustaining higher satisfaction levels. Existing research also shows that health insurance, subsidies for vulnerable groups, and charitable services are significantly associated with patients’ willingness to seek care and their satisfaction (31). Based on this analysis, we propose:

*H3*: Economic support has a positive association with PMD’ SCMHS.

## Methods

### Study context

This study was based on a cross-sectional survey conducted between January 2021 and May 2022. A stratified sampling approach was adopted, covering three Chinese cities—Shanghai, Changsha, and Liuzhou—representing high, medium, and low levels of economic development in the eastern, central, and western regions of China, respectively. Within each city, one or two counties (or districts) were selected, followed by the selection of three to four typical communities within each county/district. All chosen communities had functional health service centers or local clinics providing services to individuals with mental disorders.

### Participants and survey

Eligible participants were individuals diagnosed with mental disorders, registered in community health records, and who had received community-based mental health services for at least 1 year. The inclusion and exclusion criteria were as follows:

- Inclusion criteria: (a) diagnosed with a mental disorder, (b) registered in community health records, and (c) received community-based mental health services for at least 1 year.
- Exclusion criteria: (a) patients with severe cognitive impairment (determined based on clinical records and the judgment of experienced community health workers, as no standardized screening instrument was used in this real-world service setting) and (b) individuals under 14 years of age.

Participation was anonymous and voluntary. This study was conducted in accordance with the *Ethical Review Measures for Life Sciences and Medical Research Involving Humans* issued by China’s National Health Commission. Prior to the survey, all participants or their family guardians were informed of the study’s purpose and procedures, and verbal informed consent was obtained before data collection commenced.

The survey team, consisting of more than 10 members including professors, students, and community health workers, administered questionnaires through one-on-one interviews to ensure accurate and complete responses. A total of 583 questionnaires were collected. After applying inclusion and exclusion criteria, 508 valid samples were retained for final analysis. Table 1 presents the sample distribution and characteristics.

**Table 1:** Presents the sample distribution and characteristics (*n* = 508).

Demographic characteristics		Frequency	Scale (%)
Gender	Female	215	42.3
	Male	293	57.7
Age	Youth	84	16.6
	Middle-aged	311	61.2
	Older adults	113	22.2
Marriage	Unmarried	225	44.3
	Married	221	43.5
	Others	62	12.2
Education	Primary and below	144	28.3
	Junior high school degree	199	39.2
	High school degree	108	21.3
	Bachelor degree or above	57	11.2
Region	Eastern (Shanghai)	110	21.7
	Central (Changsha)	192	37.8
	Western (Liuzhou)	206	40.5

### Measurement

#### Dependent variable: satisfaction with community mental health services (SCMHS)

Satisfaction was measured using the question: “Overall, how satisfied are you with the community mental health services you have received?” Responses were recorded on a 5-point Likert scale: 1 = very dissatisfied; 2 = somewhat dissatisfied; 3 = neutral; 4 = somewhat satisfied, and 5 = very satisfied. Following methodologies employed in previous studies (49, 50), a binary satisfaction variable was constructed for analytical purposes. Responses indicating “somewhat satisfied” or “very satisfied” were classified as high satisfaction (coded as 1), while “neutral,” “somewhat dissatisfied,” and “very dissatisfied” responses were categorized as low satisfaction (coded as 0).

#### Independent variables: social support (SS)

Drawing on existing literature (51–54) and accounting for the specific context of this study, three types of social support were operationalized as independent variables: emotional support, technical support, and economic support. We acknowledge that single-item indicators are a limitation, though they are commonly used in large-scale mental health surveys (55, 56). Emotional Support, reflecting psychological support, was evaluated through privacy protection (PP). Technical Support was measured using rehabilitation guidance (RG). Economic Support was evaluated through reimbursement satisfaction (RS). Each dimension was captured via the question: “To what extent have you received the following types of support?” Responses were rated on a 5-point scale: 1 = very small, 2 = relatively small, 3 = moderate, 4 = relatively large, and 5 = very large. Higher scores represent higher levels of perceived emotional, technical, or economic support.

### Control variables

Consistent with prior research (14, 46, 57), individual characteristics of patients are known to influence perceptions of service quality. Therefore, age, gender, marital status, and educational attainment were included as control variables. Additionally, given that mental disorder treatment often entails long-term financial burden on households, family income was also incorporated as a control. Age, education level, and family income were treated as categorical variables, while gender and marital status were coded as binary variables.

### Statistical analysis

This study first employed one-way analysis of variance (ANOVA) to examine differences in community mental health service satisfaction across different types of social support. Subsequently, a probit regression model was used to analyze the relationship between social support and satisfaction with community mental health services.

To enhance the robustness of the findings, we pre-specified the following analytical strategies prior to conducting the analyses: (a) propensity score matching (PSM) with four matching methods (nearest neighbor, radius, caliper-based nearest neighbor, and kernel matching), using the control variables from the baseline model as covariates; (b) replacement of explanatory variables with alternative indicators (family counseling, medication guidance, and reimbursement ratio); and (c) alternative regression models (Logistic and OLS) to verify the consistency of the results.

For statistical analysis, one-way ANOVA was conducted using PASW Statistics 18, while all regression analyses and robustness tests were performed in STATA 16.0. The significance level was uniformly set at *p* < 0.05, and *p*-values are reported as *p* < 0.001 where applicable.

## Results

### One-way ANOVA results

A one-way analysis of variance (ANOVA) was conducted to examine differences in SCMHS across different types of social support (emotional, technical, and economic support). The results (Table 2) indicated that the differences in SCMHS under the three types of social support were all statistically significant (*p* < 0.001), suggesting that patients’ perceptions of different dimensions of social support are significantly associated with their SCMHS. This preliminarily confirms a significant association between social support and SCMHS.

**Table 2:** Results of one-way ANOVA.

Variables	*F*-value	Sig.
PP	10.709	<0.001
RG	13.035	<0.001
RS	15.093	<0.001

### Baseline regression results

To mitigate multicollinearity, the three independent variables were included separately in the regression models. All results are reported in the form of marginal effects (see Table 3). Emotional support (PP) showed a significant positive association with SCMHS (*β* = 0.133, *p* < 0.001), suggesting that higher recognition of privacy protection is associated with higher service evaluation. Technical support (RG) also showed a significant positive association (*β* = 0.117, *p* < 0.001), reflecting that satisfaction with rehabilitation guidance is associated with higher service evaluation. Economic support (RS) similarly had a significant positive association with SCMHS (*β* = 0.087, *p* < 0.001), suggesting that satisfaction with medical reimbursement and subsidy policies is associated with higher perceived service quality. A comparison of coefficient sizes revealed that emotional support had the strongest effect, followed by technical support, while economic support had the smallest effect.

**Table 3:** Baseline regression results.

Variables	SCMHS (0-Low; 1-High)		
	(1)	(2)	(3)
PP	0.133***(0.016)	/	/
RG	/	0.117***(0.011)	/
RS	/	/	0.087***(0.012)
Control Variables	Control	Control	Control
*P*-value	<0.001	<0.001	<0.001
Pseudo *R*^2^	0.363	0.441	0.187
Sample size	508	508	508

*** in the table denote significance at the 1% levels. The figures in parentheses are standard errors.

### Robustness tests

#### Propensity score matching (PSM) analysis

To address potential endogeneity, social support was treated as the treatment variable, SCMHS as the outcome variable, and the control variables from the baseline model as covariates. Samples were divided into high and low social support groups based on support levels. A Logit model was constructed, and the average treatment effect on the treated (ATT) was estimated using four common matching methods: nearest neighbor, radius, caliper-based nearest neighbor, and kernel matching. The results in Table 4 show that the ATT values obtained from all four matching methods were significant at the 5% level, indicating that even after controlling for endogeneity, all three types of social support remained significantly associated with patients’ SCMHS, supporting the robustness of the baseline regression results.

**Table 4:** Results of propensity score matching (PSM) analysis.

Matching methods	ATT (*T*-value)		
	PP	RG	RS
Nearest neighbor matching	0.6428^**^(7.86)	0.5874^**^(8.27)	0.2512^**^(6.17)
Radius matching	0.6394^**^(8.20)	0.5677^**^(8.41)	0.2131^**^(5.91)
Caliper matching	0.6385^**^(7.90)	0.5962^**^(8.36)	0.2598^**^(6.43)
Kernel matching	0.6376^**^(8.29)	0.5551^**^(8.57)	0.2205^**^(6.37)

For nearest neighbor matching, a 1:4 matching ratio was used. For radius matching, the caliper was set to 0.01. For kernel matching, a 1:4 matching ratio within the caliper was employed, with the caliper set to 0.01. The figures in parentheses are standard errors. ** in the table denote significance at the 5% levels.

### Replacing explanatory variables

Building on the PSM analysis, explanatory variables were replaced to further test robustness. Specifically, “Family counseling” (FC), “Medication guidance” (MG) and “Reimbursement ratio” (RR) were used to replace the original variables “Privacy protection” (PP), “Rehabilitation guidance” (RG) and “Reimbursement satisfaction” (RS) respectively. Regression results in Table 5 show that all substituted variables had a significant positive association with SCMHS, consistent with the baseline regression results, further validating a robust positive relationship between social support and SCMHS.

**Table 5:** Regression results with alternative explanatory variables.

Variables	SCMHS (0-Low; 1-High)		
	(7)	(8)	(9)
FC	0.124***(0.011)	/	/
MG	/	0 0.128***(0.014)	/
RR	/	/	0.067***(0.014)
Control Variables	Control	Control	Control
*P*-value	<0.001	<0.001	<0.001
Pseudo *R*^2^	0.437	0.380	0.099
Sample Size	508	508	508

*** in the table denote significance at the 1% levels. The figures in parentheses are standard errors.

### Changing regression models

Logistic regression and OLS models were used to replace the Probit model for additional robustness checks. Column 1 of Table 6 reports the marginal effects from the logistic regression, showing significance and direction consistent with the baseline results. Column 2 presents the OLS regression results, which also align with the baseline model in terms of significance and coefficient signs, further supporting the robustness of the finding that social support is positively associated with SCMHS.

**Table 6:** Results from logistic and OLS regression models.

Variables	Logistic model (1)	OLS model (2)
PP	0.136***(0.013)	0.243***(0.028)
RG	0.115***(0.010)	0.214**(0.021)
RS	0.089***(0.013)	0.103***(0.016)
Control variables	Control	Control
Sample size	508	508

***, ** in the table denote significance at the 1%, 5% levels, respectively. The figures in parentheses are standard errors.

### Heterogeneity analysis results

Based on the baseline regression, the associations of social support on service satisfaction across different gender and age groups were further analyzed. Columns 1 and Column 2 of Table 7 show regression results for male and female subgroups, respectively. Social support had a significant positive association with SCMHS in all genders, with coefficients for all three types of support being higher among males, suggesting a stronger association between social support and SCMHS among male patients. Columns 3, 4, and 5 present results for youth, middle-aged, and older adult groups, respectively. Social support had significant positive associations across all age groups, but the relative strength of associations varied by support type: emotional support (PP) and technical support (RG) showed the strongest associations with youth (*β* = 0.141, *p* < 0.001; *β* = 0.264, *p* < 0.001), while economic support (RS) showed the strongest association with the older adults (*β* = 0.093, *p* < 0.001). This suggests that youth are more strongly associated with emotional and technical support, whereas the older adults show a stronger association with economic support.

**Table 7:** Results of heterogeneity analysis.

Variables	SCMHS (0-Low; 1-High)				
	Male (1)	Female (2)	Youth (3)	Middle-aged (4)	Older adults (5)
PP	0.140*** (0.017)	0.113*** (0.019)	0.141*** (0.026)	0.118*** (0.017)	0.139*** (0.030)
RG	0.115*** (0.013)	0.112*** (0.016)	0.264*** (0.052)	0.109*** (0.013)	0.111*** (0.016)
RS	0.083*** (0.016)	0.082*** (0.018)	0.092*** (0.035)	0.084*** (0.015)	0.093*** (0.026)
Control variables	Control	Control	Control	Control	Control
Sample size	293	215	84	311	113

*** in the table denote significance at the 1% levels. The figures in parentheses are standard errors.

## Discussion

Based on the theory of social support, this study constructs an analytical framework comprising three dimensions—emotional, instrumental, and economic support—to explore how perceived social support is associated with PMD’ SCMHS. Using survey data from three Chinese cities—Shanghai, Changsha, and Liuzhou—the study employed a probit model to systematically investigate the associations of different dimensions of social support on SCMHS, as well as the heterogeneity of these effects across gender and age groups. The results indicate that social support is significantly and positively associated with PMD’ SCMHS overall, with notable variations in the strength of associations across different support dimensions, particularly highlighting the prominent role of emotional support. Furthermore, the association of social support on SCMHS exhibits significant heterogeneity between male and female patients, as well as between younger, Middle-aged and older age groups.

Specifically, emotional support shows a significant positive association with SCMHS, suggesting the critical role of psychological support in improving patients’ evaluation of community mental health services. This finding is consistent with existing literature (58–60) and further emphasizes the importance of protecting patient privacy and providing systematic family psychological counseling during service delivery (12, 40). Social discrimination not only exacerbates psychological pressure on patients and their families but also hinders their social integration. Patients often experience shame and stigma due to a lack of disease awareness (58) and external discrimination (59), while their families may avoid seeking help as a result, leading to social isolation. Therefore, it is plausible that service institutions’ protection of privacy may enhance patients’ psychological security, and family psychological counseling may help alleviate multifaceted pressures.

Technical support also shows a significant positive association with SCMHS, suggesting that services such as professional rehabilitation guidance and medication advice are recognized by patients and are associated with their overall satisfaction. This result aligns with the studies of Ning and Guo (10), Arya (13), and Yang et al. (14), reflecting patients’ emphasis on the professionalism and effectiveness of services. It also highlights the need for higher professional skills and ethical standards among community mental health workers.

Economic support similarly demonstrates a significant positive association with SCMHS (*β* = 0.087, *p* < 0.001), suggesting that financial assistance is associated with reduced financial burden on patients’ families and, in turn, with higher service evaluation. This finding is consistent with the conclusions of Roberts et al. (55), Liu et al. (31), and Andrade et al. (47). Mental illness often involves long treatment cycles and high costs, and caregivers may lose employment opportunities due to accompanying responsibilities, which may easily leading families into financial difficulties (44, 45). Therefore, it is plausible that increasing medical insurance reimbursement rates and establishing special subsidy policies may help alleviate economic pressure and relate to higher service satisfaction.

A comparison of the three support dimensions reveals that emotional support has the greatest association, underscoring that medical and technical services alone may be insufficient to fully account for the service experience. Instead, an integrated support model addressing psychological, social, and technical aspects may be necessary (15, 16), which aligns with the core principles of the biopsychosocial medical model.

Heterogeneity analysis across groups shows that the associations of social support on SCMHS varies by gender and age. In terms of gender, social support shows a positive association with SCMHS for both males and females, but the association is stronger for males. One possible explanation is that this may be related to traditional gender roles, where men often bear greater economic and social responsibilities (61), potentially making them more reliant on external support to restore social functioning. In terms of age differences, older adults show a stronger association with economic support, possibly because of limited financial resources and a stronger focus on medical cost-sharing. Younger individuals, however, show stronger associations with technical and emotional support, as they are in a stage of social growth, have less mature disease awareness, and may be more susceptible to social identity and peer influence (62). These findings suggest that policy-making and service design should be more targeted to meet the diverse needs of different populations, promoting a more precise and inclusive development path for mental health services.

### Contributions and limitations

The theoretical contribution of this study lies in its operationalization of social support into three quantifiable dimensions—emotional, technical, and economic—and the construction of a systematic “social support–service satisfaction” analytical framework. This provides a clear and verifiable research path for examining how social support is associated with mental health services. On a practical level, using empirical data from multiple regions in China, the study provides evidence of the significant associations of multidimensional social support with SCMHS and identifies its heterogeneous associations across gender and age groups. This offers theoretical and empirical support for implementing differentiated and precise service policies based on the biopsychosocial medical model.

However, this study has several limitations. First, the cross-sectional design limits causal inference. Although significant associations were identified, these findings do not imply causality. Endogeneity due to omitted variables or reverse causality cannot be fully excluded despite robustness checks. Longitudinal or experimental designs are needed to establish causal relationships. Second, data were collected from only three cities (Shanghai, Changsha, and Liuzhou). The sample size (*n* = 508) and geographical coverage are limited, which may affect generalizability. Future research should expand sample size and geographical scope, including rural–urban comparisons and broader heterogeneity analyses across factors such as disease type or illness duration. Third, each dimension of social support was measured using a single-item indicator (privacy protection, rehabilitation guidance, and reimbursement satisfaction), which may not fully capture the multidimensional nature of these constructs. Single-item measures are also more susceptible to random measurement error and may underestimate true associations. Future research should develop and validate multi-item scales to more comprehensively assess emotional, technical, and economic support in community mental health settings.

## Conclusion

This study draws the following conclusions: First, emotional, technical, and economic social support all show significant positive associations with SCMHS, with emotional support having the most prominent association. Second, the associations of social support with SCMHS exhibit gender and age heterogeneity. Males show stronger associations with social support; older adults show stronger associations with economic support; and younger groups show stronger associations with emotional and technical support. Overall, SCMHS is associated with multidimensional social support: emotional support relates to a foundation of trust and care, technical support relates to provides professional and effective service guarantees, and economic support relates to service accessibility and continuity. These three dimensions synergize and reinforce each other, collectively shaping patients’ service experiences through complex psychological and social mechanisms. This study provides empirical evidence and directional insights for constructing an integrated social support system and promoting precise mental health service policies.

## Data Availability

The raw data supporting the conclusions of this article will be made available by the authors, without undue reservation.
